# Heat Stress and Microbial Stress Induced Defensive Phenol Accumulation in Medicinal Plant *Sparganium stoloniferum*

**DOI:** 10.3390/ijms25126379

**Published:** 2024-06-09

**Authors:** Mengru Sang, Qinan Liu, Dishuai Li, Jingjie Dang, Chenyan Lu, Chanchan Liu, Qinan Wu

**Affiliations:** 1 Jiangsu Collaborative Innovation Center of Chinese Medicinal Resources Industrialization, Nanjing University of Chinese Medicine, Nanjing 210023, China; sangmengru@njucm.edu.cn; 2 State Key Laboratory on Technologies for Chinese Medicine Pharmaceutical Process Control and Intelligent Manufacture, Nanjing University of Chinese Medicine, Nanjing 210023, China; 3 School of Pharmacy, Nanjing University of Chinese Medicine, Nanjing 210023, China; 20210651@njucm.edu.cn (D.L.); jingjiedang@njucm.edu.cn (J.D.); 20220921@njucm.edu.cn (C.L.); 4 Nanjing Institute for Food and Drug Control, Nanjing 211198, China; liuqinan0728@163.com

**Keywords:** heat stress, microbial stress, phenol, amino acid, *Sparganium stoloniferum*, chemical defense

## Abstract

An approach based on the heat stress and microbial stress model of the medicinal plant *Sparganium stoloniferum* was proposed to elucidate the regulation and mechanism of bioactive phenol accumulation. This method integrates LC–MS/MS analysis, 16S rRNA sequencing, RT–qPCR, and molecular assays to investigate the regulation of phenolic metabolite biosynthesis in *S. stoloniferum* rhizome (SL) under stress. Previous research has shown that the metabolites and genes involved in phenol biosynthesis correlate to the upregulation of genes involved in plant–pathogen interactions. High-temperature and the presence of *Pseudomonas* bacteria were observed alongside SL growth. Under conditions of heat stress or *Pseudomonas* bacteria stress, both the metabolites and genes involved in phenol biosynthesis were upregulated. The regulation of phenol content and phenol biosynthesis gene expression suggests that phenol-based chemical defense of SL is stimulated under stress. Furthermore, the rapid accumulation of phenolic substances relied on the consumption of amino acids. Three defensive proteins, namely Ss4CL, SsC4H, and SsF3′5′H, were identified and verified to elucidate phenol biosynthesis in SL. Overall, this study enhances our understanding of the phenol-based chemical defense of SL, indicating that bioactive phenol substances result from SL’s responses to the environment and providing new insights for growing the high-phenol-content medicinal herb SL.

## 1. Introduction

Phenols, ubiquitous secondary metabolites in plants, play a vital role in plant defense mechanisms [1]. They enhance plant protection against various abiotic stresses, including reactive oxygen species [2], drought [3], and biotic stresses, like microbes [4] and herbivores [5]. *Sparganium stoloniferum* is a medicinal plant distributed in Asia, including China and Korea [6,7], known for its high tolerance to different environments [8,9,10]. It has been used in China for over 1200 years, since the Tang Dynasty, to promote blood circulation and remove blood stasis. Interestingly, phenols, such as phenolic acids and flavonoids, are important bioactive substances found in the *S. stoloniferum* rhizome (SL), exhibiting antiplatelet aggregation [11,12] and anticancer activities [13,14]. Therefore, phenols not only protect *S. stoloniferum* from stress but also contribute to its medicinal properties.

Phenolic acids and flavonoids, as pharmaceutical substances in SL, originate from the phenylpropanoid biosynthesis pathway, which begins with aromatic amino acids, such as phenylalanine and tyrosine, the final products of the shikimate pathway [15]. Therefore, amino acids serve as the foundation of phenol biosynthesis. Phenylalanine is initially converted to cinnamic acid by the deamination activity of phenylalanine ammonia-lyase (PAL, EC 4.3.1.24) [16], followed by hydroxylation and frequent methylation activity of *trans*-cinnamate 4-monooxygenase (C4H, EC 1.14.14.91) to produce *p*-coumaric acid and other acids containing a phenylpropane (C6–C3) unit [17]. Then, *p*-coumaric acid is catalyzed by 4-coumarate: coenzyme A ligase (4CL, EC 6.2.1.12) to form *p*-coumaroyl-CoA, which serves as a substrate for the biosynthesis of lignin and flavonoids [18]. The accumulation of lignin and flavonoids in plants provides mechanical strength and chemical protection against harmful elements in the environment [19]. During the process of lignin and flavonoid biosynthesis, various phenolic acids are generated to provide chemical defense [20]. Therefore, phenylalanine ammonia-lyase (PAL), trans-cinnamate 4-monooxygenase (C4H), and 4-coumarate: coenzyme A ligase (4CL) are crucial enzymes influencing the biosynthesis of phenols in plants, affecting the defense and quality of the plant. The first three steps of the phenylpropanoid pathway are also referred to as the general phenylpropanoid pathway, which is linked to both the core and specialized metabolisms and provides precursors for all downstream metabolites [20]. Apart from PAL, C4H, and 4CL, another crucial enzyme influencing flavonoid biosynthesis is flavonoid 3′,5′-hydroxylase (F3′5′H, EC 1.14.14.81), which hydroxylates the B-ring of flavonoids at the 3′- and 5′-position [21].

During the growth of SL, the phenylpropanoid biosynthesis pathway and associated genes of differentially expressed proteins (DEPs) were notably active in the early growth stage. Additionally, differential metabolites (DMs) and differentially expressed genes (DEGs) were highly enriched in phenylpropanoid and flavonoid biosynthesis, and were upregulated during this stage, indicating the critical role of phenol biosynthesis in regulating SL growth. At the early growth stage of SL, DEGs and DEPs related to plant–pathogen interactions were upregulated, resulting in the increased generation of phenolic DMs [22]. Therefore, the early growth stage is crucial, as it represents SL’s adaptation to the environment. In China, where *S. stoloniferum* grows, the plant experiences abiotic stress, particularly long-term heat stress, with temperatures exceeding 30 °C at the early growth stage of SL. Previous research revealed significantly higher expression levels of genes related to phenol biosynthesis in the heat stress group compared to the non-stress group of SL, indicating heightened phenol biosynthesis under heat stress [22]. Therefore, it is inferred that heat stress and pathogens can stimulate phenol biosynthesis to enhance SL defense. Furthermore, these stresses accompanying SL growth are significant factors contributing to the accumulation of phenolic bioactive substances in SL. The phenomenon of heat stress-induced accumulation of phenolic substances has been observed in various plant species. For instance, in *Artemisia sieberi alba*, exposure to heat stress leads to increased accumulation of secondary metabolites, including phenols, flavonoids, and proline [23]. Similarly, heat stress prompts the production of secondary metabolites, such as the tannins *Mentha piperita* and *Catharanthus roseus* [24]. Furthermore, the situation wherein pathogen stress stimulates phenol production has also been found in other plants. Activities of enzymes involved in phenol biosynthesis, such as phenylalanine ammonia lyase and polyphenol oxidase, are heightened, leading to an increase in total phenolic content in chickpeas under the stress induced by *Pseudomonas*, *Trichoderma*, and *Rhizobium* strains [4]. Therefore, it is important to assess the impact of heat stress and pathogen stress on SL.

Here, we aimed to validate the changes in phenolic metabolite content when DEGs related to plant–pathogen interactions were upregulated or when high temperatures occurred, to confirm whether heat stress and microbial stress could induce phenol accumulation in SL, resulting in higher contents of bioactive phenols. The relationships between metabolite biosynthesis and gene regulation concerning phenols were elucidated using stress and growth models of SL. Additionally, the key defense genes *C4H*, *4CL*, and *F3′5′H* from *S. stoloniferum* (*SsC4H*, *Ss4CL*, and *SsF3′5′H*) were cloned. The recombinant proteins SsC4H, Ss4CL and SsF3′5′H were expressed, and their functions were validated. Ss4CL exhibited a strong affinity for the substrate *p*-coumaric acid and efficiently catalyzed its conversion to *p*-coumaroyl-CoA. Overall, our results elucidate the phenolic defense mechanisms of SL under heat stress and microbial stress, revealing the formation mechanisms of bioactive phenols in SL. Therefore, our findings provide valuable evidence and new insights to improve the quality of the herbal medicine SL.

## 2. Results

### 2.1. Quantification of Defense Constituents in S. stoloniferum at Different Growth Stages

**Figure 1:**
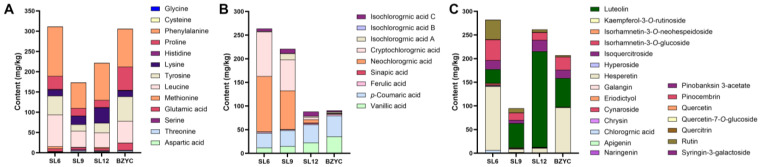
The contents of three kinds of chemical constituents in *S. stoloniferum* rhizome (SL) at different growth stages and standard SL materials (*n* = 6, the contents shown in (**A**–**C**) are the average values). (**A**) The contents of amino acids in SL at different growth stages and standard SL materials. (**B**) The contents of phenolic acids in SL at different growth stages and standard SL materials. (**C**) The contents of flavonoids in SL at different growth stages and standard SL materials. SL6, SL9, and SL12 are SL samples collected in June, September, and December, respectively. BZYC is the standard SL material, set as the control sample.

**Figure 2:**
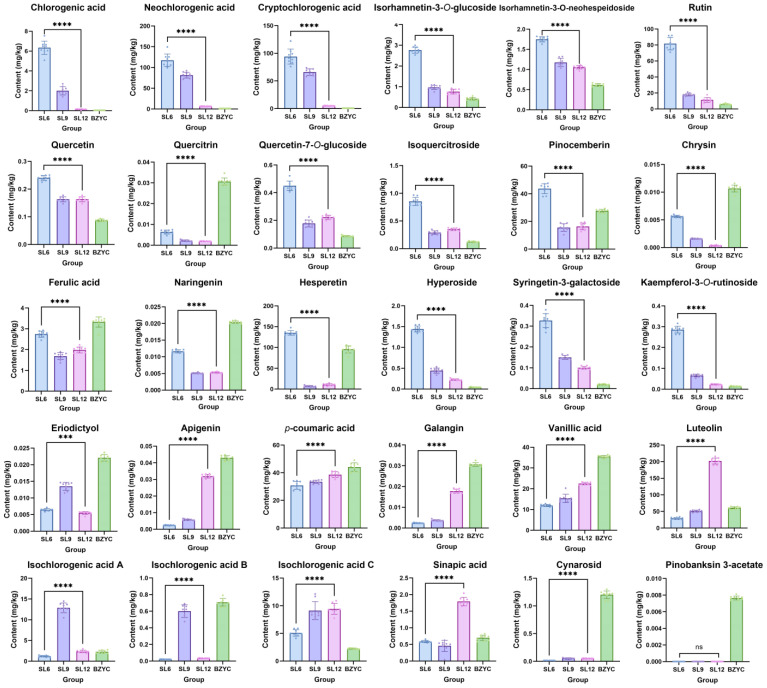
Phenol contents of SL at different growth stages and standard *S. stoloniferum* rhizome (SL) materials (*n* = 6). SL6, SL9, and SL12 are SL samples collected in June, September, and December, respectively. BZYC is standard SL material, set as control sample. The ****, ***, and ns represent *p* < 0.0001, *p* < 0.001, and *p* > 0.05 (*t*-test), respectively.

The results of amino acid and phenolic component determination in SL at different growth stages are depicted in Figure 1. A total of 30 phenolic components were detected, the total contents of three types of compounds including amino acids, phenolic acids and flavonoids were all at the highest level at the early growth stage of SL. At the early growth stage of SL, the increased accumulation of phenolic acids and flavonoids corresponds to the upregulated expression trend of genes associated with phenylpropanoid biosynthesis and plant–pathogen interactions. Notably, the total content of phenolic acids gradually decreased during growth of SL, while the change trends of amino acids and flavonoids were similar, showing a downward trend from the early to late growth stage, with the lowest contents at mid-growth stage. Details of each phenolic compound were shown in Figure 2. Except for pinobanksin 3-acetate, which was exclusively found in standard SL medicinal materials, significant differences were observed in the content of phenolic acids and flavonoids between the early and late growth stages of SL. In the early growth stage, eighteen phenolic acids and flavonoids displayed a higher content level compared to the late growth stage. Among them, twelve phenolic components, including chlorogenic acid, neochlorogenic acid, cryptochlorogenic acid, isorhamnetin-3-*O*-glucoside, isorhamnetin-3-*O*-neohesperidin, rutin, quercetin, quercitrin, chrysin, hyperoside, syringin-3-galactoside, and kaempferol-3-*O*-rutinoside, showed a gradual decrease in content as SL grew. The content of quercetin-7-*O*-glucoside, isoquercitroside, pinocembrin, ferulic acid, naringenin, and hesperetin significantly decreased from the early to late growth stages, with the content at the mid-growth stage only slightly lower than that at the late stage. Eriodictyol exhibited a significant decrease in content from the early to late growth stages, peaking during the mid-growth stage. In the late growth stage, eleven phenolic acids and flavonoids showed higher content levels, including apigenin, *p*-coumaric acid, galangin, vanillic acid, luteolin, and isochlorogenic acid C, which increased gradually as SL grew. Isochlorogenic acid A, isochlorogenic acid B, and isorhamnetin-3-*O*-glucoside showed a significant increase in content from the early to late growth stages, peaking during the mid-growth stage. The content of sinapic acid increased significantly from the early to late growth stages, with the content at the mid-growth stage only slightly lower than that at the early stage. Overall, most phenolic components had significantly higher content in the early growth stage compared to the late growth stage of SL. Additionally, neochlorogenic acid, cryptochlorogenic acid, rutin, and luteolin were found to be relatively abundant in SL. The accumulation of phenolic components and amino acids in this study is consistent with that detected in non-targeted metabolomics studies, demonstrating that phenols regulate the growth and defense of SL. The contents of three types of compounds in SL at late growth were consistent with those in standard SL material, demonstrating that the quality of the SL sample harvested in December could represent SL used in clinical applications.

**Figure 3:**
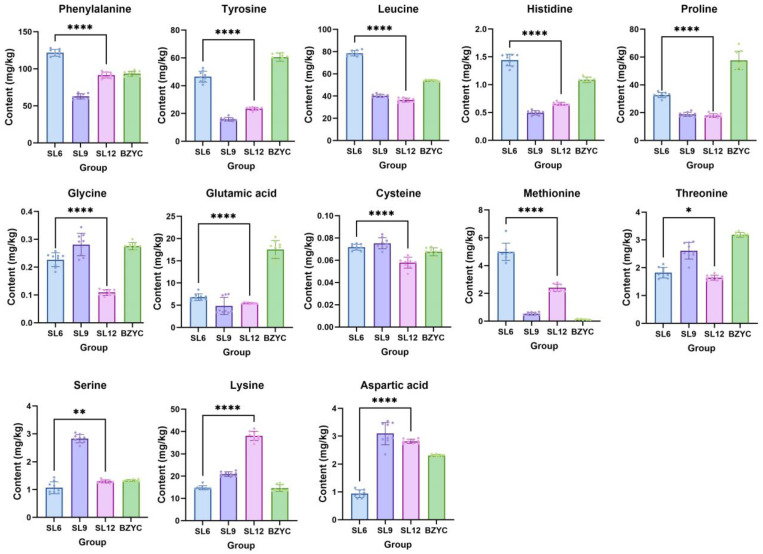
Amino acids contents of SL at different growth stages and standard *S. stoloniferum* rhizome (SL) (*n* = 6). SL6, SL9, and SL12 are SL samples collected in June, September, and December, respectively. BZYC is the standard SL material, set as the control sample. The ****, **, and * represent *p* < 0.0001, *p* < 0.01, and *p* < 0.05 (*t*-test), respectively.

The detailed results of amino acid content determination in SL at different growth stages are illustrated in Figure 3, where a total of thirteen amino acid substances were detected. Significant differences exist in the composition of various amino acids in SL between the early and late growth stages. Nine amino acids have higher levels in SL6 than in SL12, whereas only three amino acids show higher levels in the late growth stage of SL. The content of leucine and proline gradually decreases with the growth and development of SL. Phenylalanine, histidine, tyrosine, and methionine exhibit a significant decrease in content from the early to late growth stages of SL, reaching their lowest levels during growth. Glycine, cysteine, and threonine also show a significant decrease in content from the early to late growth stages of SL, peaking during the mid-growth stage. Overall, most amino acids have higher levels in the early growth stage of SL. Additionally, phenylalanine, leucine, tyrosine, and proline are considered abundant amino acids in SL. Similar to the results of phenolic component determination, the amino acid content in standard SL materials differs from the amino acid content of collected SL in this experiment, potentially influenced by other factors, such as sample collection time, production environment, and batch differences.

### 2.2. Taxonomic Identification of the Bacterial Community

**Figure 4:**
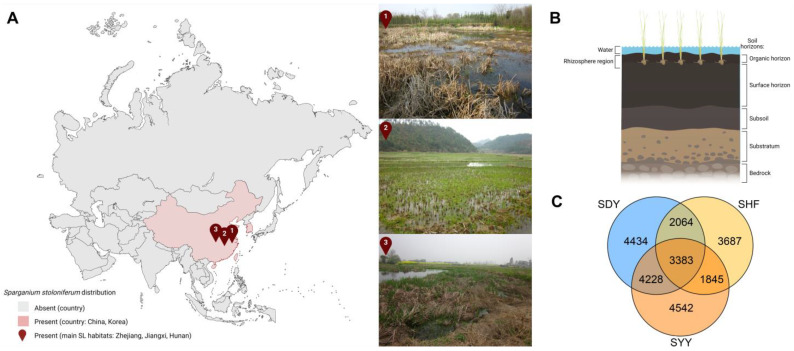
The bacterial communities in the *S. stoloniferum* rhizosphere soil from different habitats. (**A**) Distribution of countries growing *S. stoloniferum* in Aisa and the three main SL habitats in China. (**B**) The graph of *S. stoloniferum* rhizosphere soil distribution. (**C**) Venn diagram showing the operational taxonomic units of bacterial communities in the *S. stoloniferum* rhizosphere soil of three different production areas. Cartoon pictures (insets in **A**,**B**) were created with BioRender.com.

In this study, a similarity value of 0.97 was chosen as the optimal threshold for operational taxonomic unit (OTU) and taxonomic analyses. The OTUs of bacterial communities in the SL rhizosphere soil of three different production areas including Zhejiang, Hunan, and Jiangsu province, were shown in Table S1. Samples were categorized into three groups based on their growing areas (Figure 4A), and the rhizosphere soil of SL in each area was sampled (Figure 4B). Chao and ACE indices were calculated to assess microbial community abundance. Shannon and Simpson indices were utilized to evaluate the diversity of microbial community distribution (Table 1). The bacterial coverage of the samples was notably high, indicating accurate representation of bacterial communities in the rhizosphere soil of various SL habitats. Chao1 and ACE indices suggested that microbial abundance in the soil of the southern SL habitat (SHF) was relatively lower, with the lowest species richness, showing statistical significance (*p* < 0.01). Simpson and Shannon indices revealed that microbial community diversity in the soil of the central SL habitat (SYY) was significantly higher than the other two groups, showing statistical significance compared to samples from other origins (*p* < 0.01). The proportion of shared and unique bacteria in the medicinal rhizosphere soil among different regions was similar, indicating the high diversity of the soil bacterial microbiota (Figure 4C).

**Figure 5:**
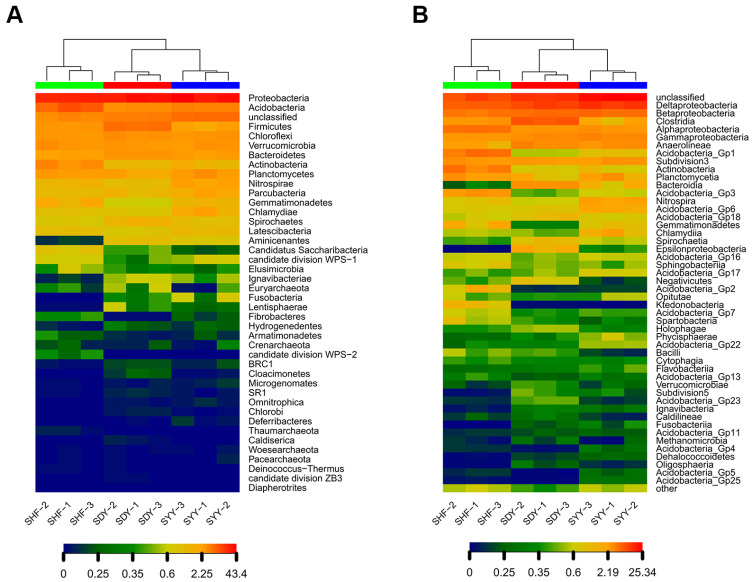
Species abundance of soil bacterial communities in rhizospheres of *S. stoloniferum* from different origins. (**A**) Heatmap of bacterial communities classified by phylum. (**B**) Heatmap of bacterial communities classified by class.

In the rhizosphere soil of the main SL habitats, a total of 741 bacterial genera belonging to 209 families, 103 orders, 78 classes, and 41 phyla were identified. The dominant bacteria in the rhizosphere soil of each production area belong to the phylum Proteobacteria, with an average abundance of 37.31%. This includes δ-Proteobacteria (15.20%), β-Proteobacteria (9.38%), and γ-Proteobacteria (4.91%), all of which rank among the top five in abundance at the class level (Figure 5). This indicates that Proteobacteria dominate the soil microbiota in different SL growth areas and are commonly present in the SL rhizosphere soil. *Pseudomonas*, a class of Gram-negative plant pathogens belonging to the family Pseudomonadaceae, order Pseudomonadales, and class Gammaproteobacteria, are widely distributed, and these infect a variety of economically important crops, making them among the most significant plant bacterial pathogens worldwide [25,26]. The result showed that *Pseudomonas* bacteria were present in the rhizospheres of the three main SL habitats, including Zhejiang, Hunan, and Jiangsu province (Table S1). Therefore, a pattern strain of *Pseudomonas syringae* was used in this study as a representative plant pathogenic bacterium that infects SL.

### 2.3. Quantification of Amino Acids and Phenols in SL under Microbial Stress and Heat Stress

**Figure 6:**
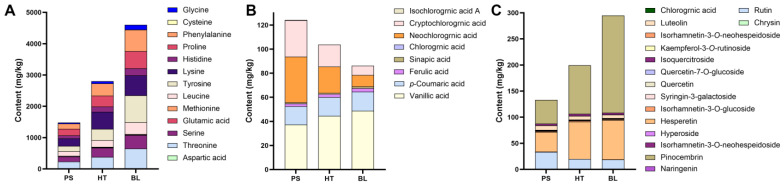
The contents of three kinds of chemical constituents in *S. stoloniferum* rhizome (SL) under microbial stress and heat stress (*n* = 6, the contents shown in (**A**–**C**) are the average values). (**A**) The contents of amino acids in SL under microbial stress and heat stress. (**B**) The contents of phenolic acids in SL under microbial stress and heat stress. (**C**) The contents of flavonoids in SL under microbial stress and heat stress. The SL sample under microbial stress was named PS. The SL sample under heat stress was named HT. The negative control group was named BL.

**Figure 7:**
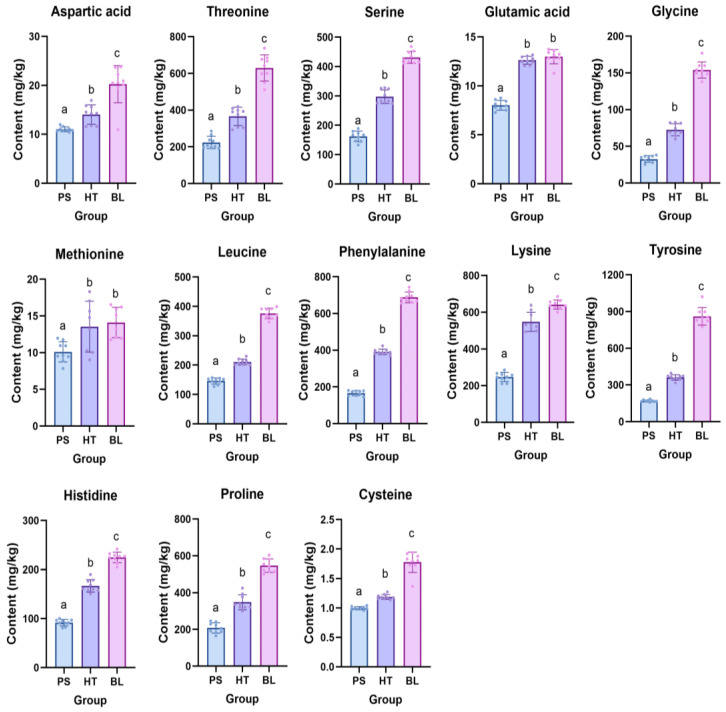
The effects of microbial stress and heat stress on the amino acid contents of the *S. stoloniferum* rhizome (SL) (*n* = 6). The SL sample under microbial stress was named PS. The SL sample under heat stress was named HT. The negative control group was named BL. The different letters (a, b, and c) next to the error bars represent statistically significant differences at *p* < 0.05 (ANOVA).

After exposure to microbial stress and heat stress, the amino acid content in SL was analyzed (Figure 6). Similar to the determination results of SL at different growth stages, the change trend of amino acids was the same with that of flavonoids. Phenolic acids accumulated more in stress groups than in the control group, contrasting with the content results of the amino acids and flavonoids. As shown in Figure 7, compared to the control group without any stress treatment, both microbial stress and high-temperature stress resulted in a decrease in the content of thirteen detected amino acids. Microbial stress had a more significant impact on amino acid content than high-temperature stress. The observed increase in amino acid content suggest that the levels of these substances decrease in SL under stress, possibly due to their transformation into other types of compounds for defense purposes.

**Figure 8:**
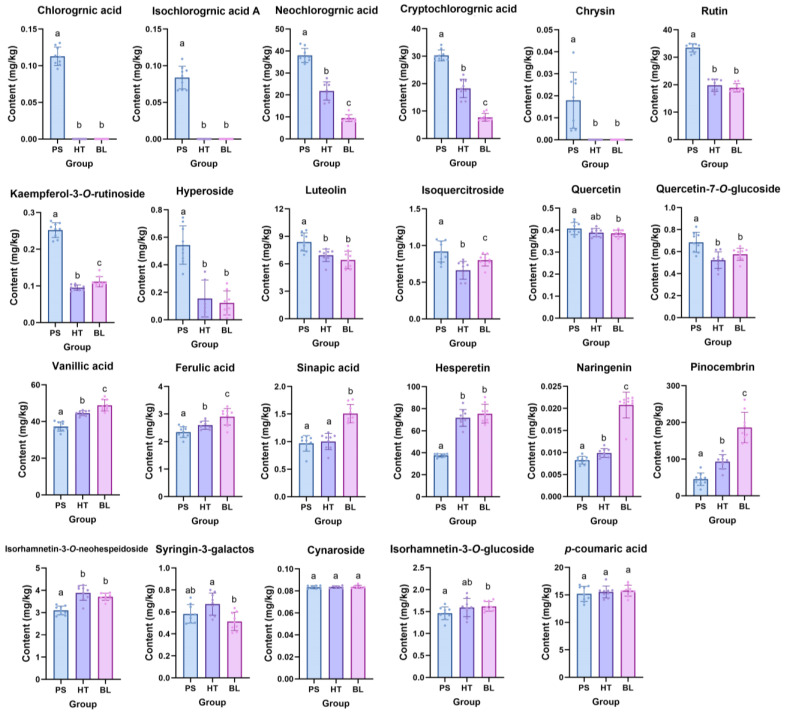
The effects of microbial stress and heat stress on the phenol contents of *S. stoloniferum* rhizome (SL) (*n* = 6). The SL sample under microbial stress was named PS. The SL sample under heat stress was named HT. The negative control group was named BL. The different letters (a, b, and c) next to the error bars represent statistically significant differences at *p* < 0.05 (ANOVA).

As shown in Figure 8, a total of 23 phenolic compounds were detected, with 13 of them displaying higher levels in SL after microbial stress compared to the control group. Among these, neochlorogenic acid, cryptochlorogenic acid, rutin, isoquercitrin, luteolin, and quercetin exhibited a trend opposite to the amino acid content in SL, with the microbial stress group showing the highest levels, followed by the high-temperature stress group, and with the control group showing the lowest levels. Additionally, chlorogenic acid, isochlorogenic acid A, and chrysin were only produced in SL under microbial stress. Nine phenolic compounds showed higher levels in SL after high-temperature stress compared to the control group. Seven phenolic compounds were detected to have decreased levels after stress in SL, among which caffeic acid, ferulic acid, sinapic acid, naringenin, hesperetin, and pinocembrin exhibited a trend similar to the amino acid content in SL. In general, most of the detected phenolic compounds in SL showed increased levels under stress, while a few showed decreased levels, and a small number of phenolic compounds did not exhibit significant changes. Among the phenolic compounds with altered levels, the alterations were more pronounced in the microbial stress group, followed by the high-temperature stress group. These results collectively indicate that both microbial stress and high-temperature stress impact the phenolic compound content in SL, with microbial stress exerting a greater impact than high-temperature stress.

### 2.4. Gene Expression under Microbial Stress

**Figure 9:**
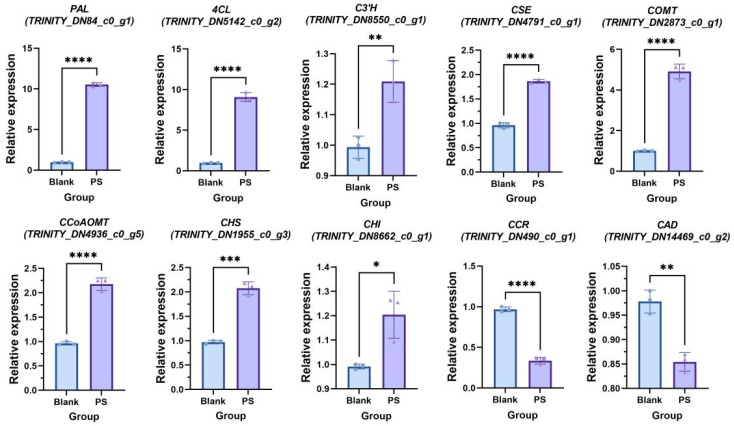
Relative expression of the gene related to lignin and phenol biosynthesis in microbial stress group and negative control group of the *S. stoloniferum* rhizome (SL) (*n* = 3). Data were normalized using *ACTIN3*. The SL sample under heat stress was named HT. The negative control group was named BL. The ****, ***, **, and * represent *p* < 0.0001, *p* < 0.001, *p* < 0.01, and *p* < 0.05 (*t*-test), respectively.

A reverse transcription–quantitative polymerase chain reaction (RT–qPCR) analysis was conducted to validate whether microbial stress influenced phenol defense or lignin defense (Figure 9). Microbial stress was assessed for their impact on the regulation of phenol and lignin synthesis. Compared to the control group without stress treatment, the expression levels of genes related to phenolic biosynthesis, such as *PAL*, *C3′H*, *CSE*, *COMT*, *CCoAOMT*, *4CL*, *CHS*, and *CHI*, were significantly higher in both the microbial stress groups. In the stress groups, the expression levels of downstream genes in the lignin biosynthesis pathway, such as *CCR* and *CAD*, were downregulated. This suggests that microbial stress may stimulate the synthesis of phenolic compounds in SL, thereby affecting the chemical defense of SL in the short term. However, microbial stress did not promote lignin biosynthesis in the short term, possibly due to the brief duration of stress, preventing plants from adjusting lignin biosynthesis to a new equilibrium. These results suggest that physical defense formation in SL is slower than the production of chemical defense.

### 2.5. Identification and Functional Analysis of Genes Related to Defensive Phenol Biosynthesis in SL

Primer pairs for *Ss4CL*, *SsC4H*, and *SsF3′5′H* were designed based on the sequences *TRINITY_DN2453_c0_g2* (NCBI: XP_020103740.1), *TRINITY_DN5418_c0_g4* (NCBI: XP_020113983.1), and *TRINITY_DN14_c0_g3* (NCBI: KAG1327666.1), respectively. The open reading frame (ORF) regions of *Ss4CL* (1623 bp), *SsC4H* (917 bp), and *SsF3′5′H* (650bp), containing *BamH* I and *EcoR* I restriction enzyme sites, were cloned from *S. stoloniferum* (Figure S1). The ORF region of *Ss4CL* encoded 540 amino acids, with a theoretical molecular mass of 58.78 kDa. Compared to *TRINITY_DN2453_c0_g2*, only 1 mutation was observed in *Ss4CL*, with the 162nd nucleotide being thymine instead of cytosine (Figure S2). However, the amino acid sequence of the Ss4CL protein’s coding region was identical to *TRINITY_DN2453_c0_g2*. The *SsC4H* gene exhibited two nucleotide mutations and a 232 bp nucleotide sequence deletion (Figure S3). The nucleotides at positions 372 and 1073 of *TRINITY_DN5418_c0_g4* were cytosine and thymine, respectively, corresponding to positions 433 and 901 in *SsC4H* as thymine and cytosine, respectively. Additionally, *SsC4H* failed to align with the nucleotide segment from positions 541 to 772 of the reference sequence. Sequencing of *SsF3′5′H* revealed a nucleotide deletion, with the thymine nucleotide at position 63 of *TRINITY_DN14_c0_g3* absent in *SsF3′5′H* (Figure S4).

**Figure 10:**
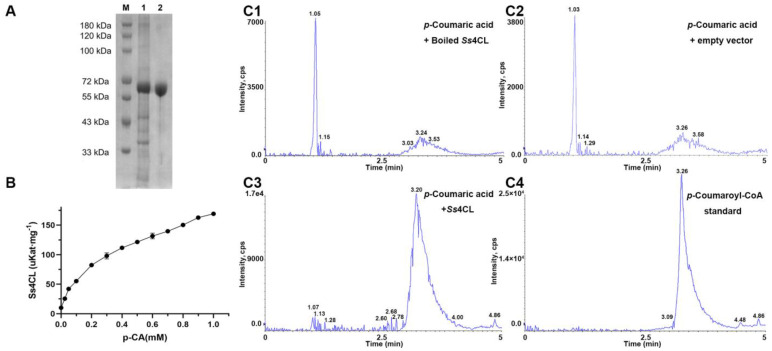
Functional characterization of phenylalanine ammonia-lyase from *S. stoloniferum.* (**A**) Purification and SDS-PAGE analysis of recombinant Ss4CL produced in *E. coli*. Lane M, 1 and 2 represent protein molecular marker, *E. coli.* harboring pET28a(+)-Ss4CL construct-induced IPTG (1 mM) at 24 h and purified recombinant Ss4CL, respectively. (**B**) Michaelis–Menten curves for the velocity of Ss4CL. Bars indicate the standard errors of the means (*n* = 3). (**C**) LC–MS/MS analysis of *p*-coumaroyl-CoA produced by a 4CL reaction using recombinant Ss4CL. (**C1**–**C4**) are extracted ion chromatograms of the *p*-coumaroyl-CoA of samples.

To confirm that the isolated cDNA encoded catalytically active Ss4CL, functional analysis was conducted using the recombinant protein expressed in *Escherichia coli*. Recombinant Ss4CL was purified using the His-tag affinity column (Figure 10A). In vitro enzymatic assays were performed using the purified recombinant Ss4CL (Figure 10B), and *p*-coumaroyl-CoA, produced by the deamination of *p*-coumaric acid, was detected by liquid chromatography–mass spectrometry (LC–MS/MS) analysis (Figure 10C). Additionally, negative control reactions using boiled recombinant Ss4CL proteins and a pET28a vector showed no product generation. The enzyme kinetic parameters of Ss4CL were determined using ultraviolet spectrophotometry and calculated using the Michaelis–Menten equation. *K*m, *V*max, *K*cat, and *K*cat/*K*m values were 1.28 mmol·L^−1^, 7.34 nKat·mg^−1^, 733.68 s^−1^, and 575.04 s^−1^·M^−1^, respectively.

**Figure 11:**
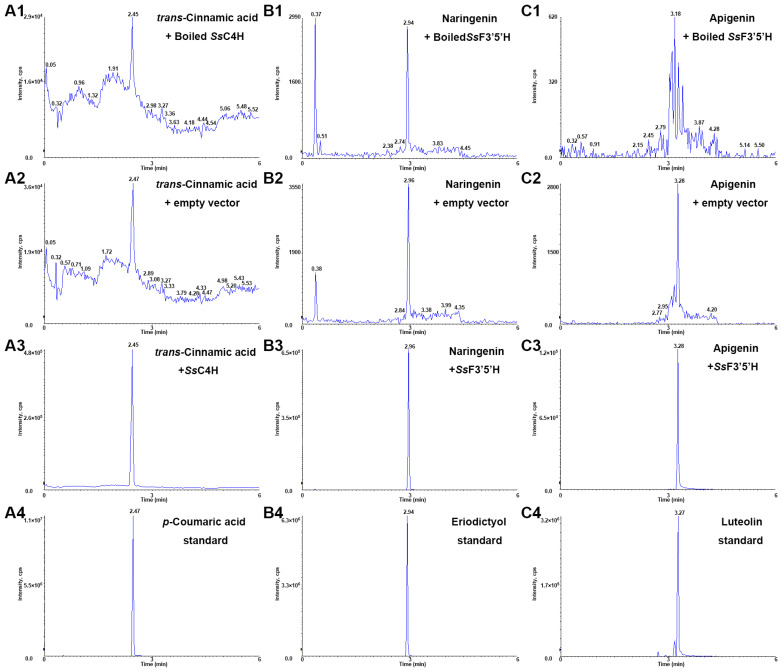
Functional identification of the recombinant SsC4H and SsF3′5′H. (**A**) LC–MS/MS analysis of *p*-coumaric acid produced by a C4H reaction using recombinant SsC4H. (**A1**–**A4**) are extracted ion chromatograms of *p*-coumaric acid of samples. (**B**) LC–MS/MS analysis of eriodictyol produced by a F3′5′H reaction using recombinant SsF3′5′H. (**B1**–**B4**) are extracted ion chromatograms of the eriodictyol of samples. (**C**) LC–MS/MS analysis of luteolin produced by a F3′5′H reaction using recombinant SsF3′5′H. (**C1**–**C4**) are extracted ion chromatograms of the luteolin of samples.

SsC4H and SsF3′5′H belong to the P450 protein family, making them more suitable for eukaryotic expression systems. Consequently, they were recombined with the pYeDP60 vector and expressed in the yeast strain WAT11. Enzyme activity verification of SsC4H and SsF3′5′H was conducted by substrate feeding. The experiment comprised an experimental group and two negative control groups, which were comprised of boiled recombinant proteins and empty vector proteins. Upon adding *trans*-cinnamic acid to the *SsC4H*-pYeDP60-WAT11 culture, hydroxylation occurred to produce *p*-coumaric acid, while neither negative control group produced *p*-coumaric acid (Figure 11A). Similarly, upon adding naringenin and apigenin separately to the *SsF3′5′H*-pYeDP60-WAT11 culture, the experimental groups produced hydroxylated products, namely eriodictyol and luteolin, whereas both negative control groups did not generate the corresponding products (Figure 11B,C).

### 2.6. Structure Prediction of Ss4CL, SsC4H, and SsF3′5′H and Molecular Docking Studies

**Figure 12:**
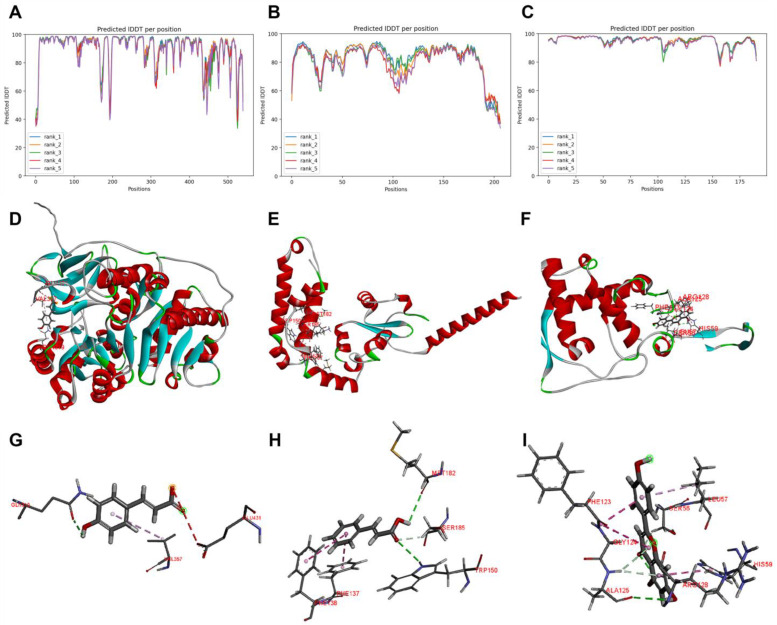
Overall structure and molecular docking of Ss4CL, SsC4H, and SsF3′5′H from *S. stoloniferum*. The predicted lDDT values of each residue position of five structure rank models of Ss4CL (**A**), SsC4H (**B**), SsF3′5′H (**C**). (**D**) Predicted structure of Ss4CL and binding site of *p*-coumaric acid and Ss4CL. (**E**) Predicted structure of SsC4H and binding site of *trans*-cinnamic acid and SsC4H. (**F**) Predicted structure of SsF3′5′H and binding site of apigenin and SsF3′5′H. (**G**) 3D diagrams show receptor–ligand interactions between *p*-coumaric and Ss4CL. (**H**) 3D diagrams show receptor–ligand interactions between *trans*-cinnamic acid and SsC4H. (**I**) 3D diagrams show receptor–ligand interactions between apigenin and SsF3′5′H.

To elucidate the molecular basis of the docking interactions between Ss4CL with *p*-coumaric acid, SsC4H with *trans*-cinnamic acid, and SsF3′5′H with apigenin, we predicted the structures of Ss4CL, SsC4H, and SsF3′5′H using the respective ligands. Five structural models for each enzyme were generated using Alphafold2 (Figure 12A–C). These models provided atomic coordinates and confidence estimates for each residue, with scores ranging from 0 to 100, where higher scores indicate higher confidence levels. The confidence measure utilized, known as the predicted local distance difference test (pLDDT), assessed the quality of the models. Most positions in the predicted models exhibited high pLDDT values. Finally, the model ranked 4 for Ss4CL, the model ranked 1 for SsC4H, and the model ranked 1 for SsF3′5′H were selected for docking simulations with *p*-coumaric acid, *trans*-cinnamic acid, and apigenin, respectively (Figure 12D–F).

Motif and conserved domain analyses of Ss4CL and its homologous proteins were conducted using TBtools software (version v2.096). Ten distinct motifs were identified (Figure S5A), and their corresponding sequence information was detailed in Table S2. These proteins universally contained motifs 1, 3, 7, 8, and 10. Notably, motifs 8 and 10 were situated within the C-terminal AMP-binding enzyme domain, spanning from Glu^453^ to Lys^528^ in Ss4CL. Acetyl-CoA synthetase contains a small structural domain with a β-folded core region in the middle and α-helices on both sides [27]. The N-terminus of the AMP binding region of Ss4CL encompassed the AMP-dependent synthetase domain, ranging from Glu^343^ to Leu^402^. Within this region, a conserved Pro-Lys-Gly triad was identified in the Ser/Thr/Gly-rich domain. Structural similarities were observed between Ss4CL and the enzymes involved in coenzyme A binding, such as long-chain fatty acyl-CoA synthetases and acetyl-CoA synthetases [28,29,30]. These enzymes belong to the ANL superfamily and are involved in catalyzing the initial adenylation of carboxylic acids, forming an acyl-AMP intermediate, followed by a second partial reaction, most commonly the formation of thioesters. These adenylation enzymes can adopt two different conformations by utilizing a 140° rotation of the C-terminal domain for adenylation and sulfurization reactions. Therefore, by using those two different conformations of the C-terminal domain, these enzymes carry out two distinct catalytic steps [31,32]. Ss4CL may have similar protein functions due to its highly similar sequence structure features, as revealed by structural domain analysis. Moreover, both Ss4CL and most homologous proteins shared the PLN02246 *p*-coumaroyl-CoA structure domain (Figure S5B) indicating their membership in the 4CL family of proteins. This family plays a crucial role in the phenylpropanoid metabolic pathway, influencing lignin and phenolic compound synthesis, with products serving as precursors for *p*-coumaroyl-CoA, a key intermediate. As shown in Figure 12G, traditional hydrogen bonding interactions were observed between *p*-coumaric acid and Ss4CL at the binding site. Specifically, Gln^319^ of Ss4CL formed hydrogen bonds with the hydroxyl group (-OH) of *p*-coumaric acid, while the phenyl ring of ferulic acid interacted electrostatically with Val^357^, contributing a negative charge to the π orbital of *p*-coumaric acid. Additionally, Glu^431^ was predicted to attract and interact with negatively charged oxygen ions.

Motif and conserved domain analyses of SsC4H and its homologous proteins were conducted using TBtools software. Ten distinct motifs were identified (Figure S6A), with their corresponding sequence information detailed in Table S3. Notably, SsC4H and its homologs contained motifs 1, 3, 8, and 10. SsC4H is classified within the cytochrome P450 enzyme family protein, exhibiting relatively low sequence conservation but highly conserved structural folding. The conserved core region comprises a meander helix, four α-helices, helices J and K, and two sets of β-folds, including the absolutely conserved EXXR motif [33]. In SsC4H, this motif spans from Glu^124^ to Arg^127^, within the Motif 1 structural domain. The sequence of SsC4H from Met^1^ to Phe^190^ is annotated to the *trans*-cinnamic acid 4-monooxygenase family, featuring typical cytochrome P450 enzyme E-class Group I structural characteristics from Arg^63^ to Va^l81^ and Leu^86^ to Phe^107^, exhibiting typical cytochrome P450 enzyme E-class Group I structural features. Through conservative domain analysis, it was found that SsC4H and its homologs belong to the cytochrome P450 superfamily (Figure S6B). As shown in Figure 12H, at the binding site of *trans*-cinnamic acid and SsC4H, two types of hydrogen bonds were observed: traditional and carbon–hydrogen bonds. Specifically, Trp^150^ and Met^182^ formed traditional hydrogen bonds with the C=O and -OH of t*rans*-cinnamic acid, respectively, while Ser^185^ provided H^+^ to the carboxyl group C=O of *trans*-cinnamic acid, forming a carbon–hydrogen bond connected to the C=O group. Additionally, the phenyl ring π orbitals of *trans*-cinnamic acid interacted hydrophobically with the alkyl groups of Phe^137^ and Pro^138^.

Motif and conserved domain analysis of SsF3′5′H and its homologous proteins were performed using TBtools software. Ten distinct motifs were identified (Figure S7A), with their corresponding sequence information detailed in Table S4. Notably, SsF3′5′H and its homologs contained motifs 1, 2, 3, and 7. Similar to SsC4H, SsF3′5′H also belongs to the cytochrome P450 enzyme family. The conserved structural domain of SsF3′5′H, spanning from Phe^123^ to Gly^132^, is located within the Motif 1 domain, surrounding a highly conserved cysteine residue. Specific regions of SsF3′5′H, including Gln^40^ to Pro^58,^ Asn^81^ to Ser^105^, and Leu^120^ to Cys^130^ in SsF3′5′H exhibit typical cytochrome P450 enzyme E-class Group I structural features. Conservation domain analysis revealed that SsF3′5′H and its homologs are classified under the cytochrome P450 superfamily (Figure S7B). As shown in Figure 12I, the molecular interactions between apigenin and SsF3′5′H were examined, revealing the presence of traditional hydrogen bonds, carbon–hydrogen bonds, and hydrophobic interactions. For instance, the hydroxyl group at C7 of apigenin forms a traditional hydrogen bond with Ala^125^, while Ala^125^ forms carbon–hydrogen bonds with rings A and C. Additionally, Ser^56^ forms a carbon–hydrogen bond with ring C at position C3. The hydrophobic interactions include π-orbital interactions of ring C in apigenin with the alkyl group of Leu^57^, as well as edge-to-face interactions of Phe^123^ with rings B and C. His^59^ exhibits amide-π stacking with ring A, and Gly^124^ interacts with apigenin molecules through van der Waals forces.

## 3. Discussion

Previous research indicated that DEGs related to plant–pathogen interaction pathways were upregulated when the phenol-based chemical defense was active at the early growth stage of SL [22]. To validate the role of phenols in the growth and defense mechanisms of SL and the conclusion that phenols are synthesized as a chemical defense mechanism during the early growth stage of SL, targeted LC–MS/MS metabolomics was employed to detect phenol and amino acid content in SL at different growth stages. The determination results are consistent with that observed in untargeted LC–MS/MS metabolomics, indicating notably higher levels of phenolic acids and flavonoids during the early growth stage compared to later stages. Similarly, amino acids, such as phenylalanine, leucine, and proline, known for their role in plant stress response [34,35,36], exhibited higher levels during the early growth stage of SL. Phenylalanine, the most abundant amino acid in SL, serves as a precursor to phenylpropanoid biosynthesis [20], indicating active phenylpropanoid pathway activity in SL. Phenylalanine undergoes PAL deamination to produce *trans*-cinnamic acid [16], which further undergoes C4H oxidation and 4CL coenzyme A linkage, generating a series of lignin phenol precursors that are then transformed into diverse phenolic components by various phenol synthesis enzymes [15]. Phenolic components play a crucial role as chemical defense agents in plants [2,4], with phenylalanine serving as a key defensive amino acid component in SL. Content analysis revealed that phenylalanine is the predominant amino acid component in SL, and both phenylalanine and most phenolic components exhibit higher levels during the early growth stage of SL compared to later stages, suggesting a greater reliance on phenolic compounds for chemical defense during early growth stages. Numerous studies have shown that many amino acids stored in plants are susceptible to various abiotic stresses [36,37]. Under stress conditions, plants experience elevated levels of proline and other amino acids [23,35]. Proline accumulation serves as an osmoprotectant, shielding plants from adverse environmental conditions, such as low and high temperatures, salinity, UV radiation, and osmotic stress [34,38]. Alanine enhances plant resistance to stress, boosts adaptability to environmental challenges, regulates the antioxidant system, and mitigates stress-induced damage, thereby bolstering overall resistance [39]. The content of alanine and proline, amino acids abundant in SL, decreased as SL grew, suggesting a requirement for chemical defenses in the early growth stages to support the plant’s defense function. This observation supports the conclusion that SL primarily relies on chemical defense mechanisms during its early growth stages, alongside alterations in phenolic composition. Phenolic compounds, known for their pharmacological activity in SL [11,12,13,14], accumulate during the development of the medicinal herb, exhibiting a distinct pattern of content accumulation and gene regulation. The growth environment is complex, yet few diseases occur in SL, which are closely linked to its defense mechanisms. A primary mechanism for plants to combat stress involves reinforcing cell walls to create a physical barrier, bolstering resistance to abiotic stress [40] and biotic stress [4,41,42]. During the early growth stages of SL, characterized by low tissue hardness and high demand for lignin biosynthesis, the phenylpropanoid synthesis pathway is activated to produce lignin phenolic precursors with chemical defense properties, compensating for the lack of physical defense. Results from transcriptomic, proteomic, non-targeted metabolomic, and targeted metabolomic analyses collectively confirm the activation of phenolic chemical defense mechanisms during the early growth stages of SL [22]. With the growth of SL, the levels of phenolic substances and defensive amino acids decline significantly, highlighting the combined defense mechanisms involving physical reinforcement of cell walls through lignin and the chemical defense of SL. The need for lignin synthesis results in the production of lignin phenolic precursors, serving as defensive compounds in SL and active ingredients in medicinal herbs, exhibiting pharmacological effects, such as anticoagulant and anticancer properties [11,12,13,14].

In response to rapid environmental changes, genes involved in the phenylpropanoid biosynthesis pathway are rapidly upregulated, leading to the accumulation of phenolic compounds [43]. Studies have shown that exposure of plants to stress induces an increase in the production of phenolic compounds, which serve as defensive responses against pathogens under stress conditions [44]. The results of this study are consistent with previous findings, indicating that the rapid upregulation of phenol biosynthesis genes is a plant response to adverse environmental factors, particularly as a crucial defense strategy against biotic and abiotic stress. Following microbial and heat stress treatments, changes in phenolic compounds were accompanied by the consumption of amino acid compounds. These changes were more significant in the microbial stress group compared to the heat stress group, suggesting that amino acid compounds are likely consumed and transformed after stress, providing a material basis for plant resistance. The response of plants to microbial stress was stronger than that to heat stress, indicating a higher intensity of the chemical defense response is needed to supplement the relatively weaker physical defense against microbial stress. Microbial stress directly impacts the rhizosphere soil and water environment, while heat stress affects the entire plant through air temperature regulation, with a weaker direct impact on water-growing plants compared to microbial stress. Phenols detected in this study included phenolic acids and flavonoids, with phenolic acids and flavonoids both at high levels in SL at the early growth stage, which is sensitive to the environment, indicating that phenols are crucial for SL to respond to the environment. However, the accumulation of phenolic acids and flavonoids was different when SL was exposed to heat stress or microbial stress, while the genes related to phenolic acids and flavonoid biosynthesis, like *PAL*, *4CL*, *C3′H*, *CSE*, *COMT*, *CCoAOMT*, and *CHS*, were all upregulated. Gene expression could show a delayed effect on the eventual synthesis of secondary metabolites. According to previous research, post-transcriptional and post-translational mechanisms play roles in regulating SL growth [22]. Furthermore, a similar phenomenon appeared in other plants, and a low correlation was shown between relative contents of monoterpene secondary metabolites and the expression of related genes in *Agastache rugosa* due to detected genes located in the middle to upper reaches of the monoterpene biosynthesis pathway; the expression of these genes often has a lagging effect on the final formation of secondary metabolites [45]. An asynchronous relationship existed between the gene expression level and relative contents in *Nepeta tenuifolia*; genes showed different patterns to related metabolites. For instance, *IPR* gene showed a sinusoidal expression pattern; however, the relative content of the direct product (2*S*, 5*S*)-isopulegone decreased gradually. The accumulation of (−)-pulegone dropped sharply and (+)-menthone showed a steep increase, but the expression of *NtPR* gene was relatively low at this time point [46]. Our results were identical to those of previous studies. In this study, most of the genes selected for the RT–qPCR analysis are involved in the phenylpropanoid pathway, which is upstream of the flavonoid synthesis pathway, sharing the general phenylpropanoid pathway. The *CHS* and *CHI* genes are genes located at the upstream of flavonoid synthesis pathway; therefore, the contents of flavonoids in SL under heat stress and microbial stress differed from the expression level of related genes. Based on the expression level of phenol biosynthesis related genes and the accumulation of phenolic metabolites during growth, it is still clear that phenol could be biosynthesized by SL in response to heat stress and microbial stress.

In previous research, when SL were exposed to heat stress, genes associated with phenol biosynthesis, such as *PAL*, *4CL*, *C3′H*, *CSE*, *COMT*, *CCoAOMT*, and *CHS*, were upregulated briefly, while downstream genes related to lignin biosynthesis, *CCR*, and *CAD*, were downregulated [22]. In this study, under microbial stress, the expression of genes related to phenol biosynthesis and downstream genes associated with lignin biosynthesis mirrored those observed under heat stress, indicating that both microbial and heat stresses can induce the synthesis of phenolic compounds in SL, affecting its chemical defense in the short term. This phenomenon does not only occur in SL; for example, in chickpea, the activity of proteins involved phenol biosynthesis was upregulated after microbial stress [4]. Rapid upregulation of genes in the phenylpropanoid pathway and the accumulation of phenolics can be observed in response to environmental stress [43]. LC–MS/MS analysis results also supported the hypothesis by confirming an increase in phenolic content in stressed SL. However, neither microbial nor heat stress promoted lignin biosynthesis at the gene level in the short term, possibly because the stress duration was too brief for SL to adjust lignin biosynthesis to a new equilibrium. This also suggests that the rate of physical defense formation in SL is slower compared to chemical defense. Therefore, the formation of a plant’s physical defense is relatively slow, whereas chemical defense can rapidly occur in response to stress.

SL synthesizes phenolic substances as a chemical defense mechanism, forming the foundation of its medicinal properties. The biosynthesis of phenolic compounds necessitates the general phenylpropanoid pathway, facilitated by PAL, C4H, and 4CL enzymes. These enzymes undergo significant upregulation during early SL growth, as well as under microbial and heat stress conditions, crucially contributing to phenolic substance synthesis. The catalytic function of Ss4CL in converting phenylalanine to *trans*-cinnamic acid has been confirmed. To assess the function of Ss4CL, heterologous expression was performed in *E. coli*. BL21 (DE3) cells. Enzyme kinetics analysis revealed that, under optimal conditions, Ss4CL exhibits a high substrate affinity for *trans*-cinnamic acid with a *K*m of 1.28 mmol·L^−1^. Ss4CL catalyzes the formation of cinnamoyl-CoA from *trans*-cinnamic acid and CoA. 4CL is a key enzyme in the phenylpropanoid biosynthesis pathway, catalyzing the formation of cinnamoyl-CoA, *p*-coumaroyl-CoA, caffeoyl-CoA, feruloyl-CoA, 5-hydroxyferuloyl-CoA, and sinapoyl-CoA from cinnamic acid, *p*-coumaric acid, caffeic acid, ferulic acid, 5-hydroxyferulic acid, and sinapic acid, respectively [47]. These compounds serve as precursors in lignin biosynthesis, undergoing subsequent conversions into corresponding aldehydes and alcohols catalyzed by COMT, CCoAMOT, CCR, and CAD enzymes. The synthesis of *p*-coumaroyl-CoA from *trans*-cinnamic acid serves as a shared precursor for three monolignols and flavonoids, highlighting the role of 4CL in influencing their synthesis and contributing significantly to SL defense mechanisms and the production of medicinal phenolic compounds. Under optimal conditions, the *V*max value for the *p*-coumaric acid reaction catalyzed by Ss4CL was 7.34 nKat·mg^−1^, higher than those for Sm4CL1 and Sm4CL2 from *Salvia miltiorrhiza* [18].

The C4H protein family plays a crucial role in the phenylpropanoid biosynthesis pathway in plants, catalyzing the conversion of *trans*-cinnamic acid to *p*-coumaric acid. It initiates the first oxidation step in the phenylpropanoid biosynthesis pathway, preceding the 4CL enzyme reaction. F3′5′H is crucial in flavonoid synthesis, catalyzing hydroxylation reactions at the 3′ and 3′ and 5′ positions on the B-ring of flavonoids [21]. Because of its diverse substrates and resulting product variety, it plays a crucial role in flavonoid synthesis. Considering that both SsC4H and SsF3′5′H belong to the cytochrome P450 family, eukaryotic expression systems are preferable for their expression. We selected the yeast WAT11 for expressing SsC4H and SsF3′5′H proteins. However, since SsC4H and SsF3′5′H were localized in the microsomes, it is difficult to purify recombinant SsC4H and SsF3′5′H, so we conducted substrate-fed enzyme activity experiments. Enzyme activity experiments with Ss*C*4H and SsF3′5′H proteins revealed that SsC4H efficiently catalyzes the conversion of *trans*-cinnamic acid to *p*-coumaric acid, while SsF3′5′H hydroxylates naringenin and eriodictyol to produce apigenin and luteolin, respectively.

This study demonstrates that both heat stress and microbial stress induce the production of phenolic compounds in SL for chemical defense. The mechanism of phenolic defense production is analogous to the chemical defense mechanism employed by SL during the early growth stage. Research has demonstrated that the phenolic chemical defense barrier forms more rapidly in SL compared to physical defense mechanisms based on lignin. This suggests that the accumulation of secondary metabolites serves not only as a plant adaptation to the environment but also as a defense strategy employed by the plant itself. Pharmacologically active phenolic substances produced as a result of SL’s defense physiological activities confer medicinal value, rendering it a natural herb abundant in phenolic compounds. This offers novel insights into cultivating high-quality SL medicinal materials.

## 4. Materials and Methods

### 4.1. LC–MS/MS Analysis of SL at Different Growth Stages

The SL samples at different growth stages for LC–MS/MS analysis were *S. stoloniferum* rhizome obtained from the botanical garden of Nanjing University of Chinese Medicine in June, September, and December 2021. Healthy *S. stoloniferum* plants were selected and cleaned using ddH_2_O, and then the *S. stoloniferum* rhizomes were cut into 5 mm pieces, dried at 40 °C for 48 h, and ground into powder, before being named SL6, SL9, and SL12. Standard SL material was bought from Solarbio Science & Technology Co., Ltd., Beijing, China, named BZYC, and set as the control sample. An accurate weighing of 2 g of solid sample was performed, followed by extraction of metabolites using a solution (methanol:water, 4:1, *v*/*v*). The mixture was extracted by refluxing extraction for 1 h, and the solvent was removed by rotary steaming and dried by nitrogen blowing. The precipitation was redissolved in methanol to a final volume of 5 mL and was then filtered by a 0.22 μm nylon membrane. The supernatant and the supernatant diluted 20-fold were ready for LC–MS/MS analysis.

Information on the *p*-coumaric acid standard substances is shown in Table S5. Apart from the amino acid mixed solution standard substance, all other standard substances were prepared with 50% methanol to form a 1 mg·mL^−1^ single standard solution. Then, accurately, 100 μL of the 1 mg/mL single standard solution was taken and made up to 10 mL with 50% methanol, resulting in a 10 μg·mL^−1^ mixed standard solution A. Accurately, 1 mL of the 100 μg·mL^−1^ amino acid mixed solution standard substance mother liquor was mixed with 9 mL of 50% methanol to obtain a 10 μg·mL^−1^ mixed standard solution B. Accurately, 100 μL of mixed standard solution A and 100 μL of mixed standard solution B were combined with 800 μL of 50% methanol to produce a 1 μg·mL^−1^ mixed standard solution C. The concentrations mentioned above were theoretical concentrations. The actual concentrations of the standard substance configuration information and the mixed standards are shown in Tables S6 and S7. Due to the inclusion of phenylalanine during single standard weighing and the presence of phenylalanine in the amino acid mixed standard substance, the concentration of phenylalanine in mixed standard solution C is 2.6917 μg·mL^−1^.

The instrument platform for LC–MS/MS analysis consisted of the AB Sciex ExionLC™ AD UHPLC and AB Sciex Qtrap 5500 system. The sample was separated using an Eclipse Plus C18 column (4.6 mm I.D. × 100 mm, 3.5 μm) and then entered into mass spectrometry detection. The mobile phases consisted of methanol (solvent A) and 0.1% formic acid in 5 mM ammonium acetate (solvent B). The solvent gradient changed according to the following conditions: from 0 to 4 min, 91% B; from 4 to 6 min, 91% B to 79% B; from 6 to 10 min, 79% B to 65% B; from 10 to 12 min, 65% B to 62% B; from 12 to 16 min, 62% B to 54% B; from 16 to 20 min, 54% B to 36% B; from 20 to 21 min, 36% B to 5% B for equilibrating the systems. The sample injection volume was 5 µL and the flow rate was set to 0.8 mL·min^−1^. The column temperature was maintained at 40 °C. During the period of analysis, all samples were stored at 4 °C. Three technical replicates were used. A spectrometer equipped with an electrospray ionization (ESI) source operated in either positive or negative ion mode. The optimal conditions in positive ion mode were set as follows: CUR 35 psi, TEM 600 °C, GS1 55 psi, GS2 55 psi, ISVF 4500 V, CAD 9 psi, EP −10 V. The optimal conditions in negative ion mode were set as follows: CUR 35 psi, TEM 600 °C, GS1 55 psi, GS2 55 psi, ISVF −4500 V, CAD 9 psi, EP −10 V. The mass spectrometry parameters for all tested compounds after optimization are shown in Table S8.

Data were analyzed using SCIEX OS-Q software (Version 1.4.0.18067). Three biological replicates were used. A *t*-test was conducted for the comparison of groups and a *p* value ≤ 0.05 was regarded as statistically significant. Statistical analyses were performed, and graphs were generated using GraphPad Prism software (version 9.0).

### 4.2. DNA Extraction, PCR Amplification and 16S rRNA Sequencing

Three SL growth areas were selected, including the eastern area (Dongyang City, Zhejiang Province, E 120°19′12″, N 29°16′48″, altitude 85 m), the southern area (Shangrao City, Jiangxi Province, E 117°40′12″, N 28°30′36″, altitude 235 m), and the central area (Yueyang City, Hunan Province, E 112°30′36”, N 28°31′12″, altitude 28 m). Each sampling site was divided into three 5 m^2^ plots with 10 m intervals, and three replicates were taken. Soil samples were collected using a soil sampler at five points within each plot following the diagonal five-point method. The soil was sampled at a depth of 5–10 cm below ground and 0–20 cm near SL, mixed uniformly, and one-fifth of the mixture was taken as a single soil sample. Soil samples collected from Dongyang City, Shangrao City and Yueyang City were named SDY, SHF, and SYY, respectively. A total of 200 mg of each soil sample was taken, placed in a sterile tube, mixed with 1 mL 70% ethanol, centrifuged at 10,000× *g* at room temperature for 3 min, and then the upper liquid was discarded. PBS solution was added and centrifuged at 10,000× *g* at room temperature for 3 min, and then the upper liquid was discarded. The tube was inverted for 1 min until no liquid flowed out. The sample tube was placed in a 55 °C oven for 10 min. DNA extraction was carried out using the E.Z.N.A™ Mag-Bind Soil DNA Kit (Omega, New York, NY, USA), and the soil microbial genomic DNA was quantified precisely using the Qubit 3.0 DNA quantitation assay kit (Life, Lewisburg, PA, USA, USA).

The primers used in PCR were the V3-V4 universal primers of the Miseq sequencing platform: 341F primer—CCCTACACGACGCTCTTCCGATCTG (barcode) CCTACGGGNGGCWGCAG and 805R primer—GACTGGAGTTCCTTGGCACCCGAGAATTCCAGACTACHVGGGTATCTAATCC. The PCR reaction system was 30 μL, with the components in each tube being as follows: DNA template 20 ng, 2×Taq Master Mix 15 µL, Bar-PCR primer F (10 μM) 1 μL, primer R (10 μM) 1 μL, finally made up to 30 µL with water. The amplification program was as follows: 94 °C, 3 min; 94 °C for 30 s, 45 °C for 20 s, 65 °C for 30 s, 5 cycles; then 94 °C for 20 s, 55 °C for 20 s, 72 °C for 30 s, 20 cycles; finally 72 °C for 5 min. The second round of PCR introduced Illumina bridge PCR-compatible primers. The second round PCR reaction system was 30 μL, with the components in each tube being as follows: DNA template 20 ng, 2×Taq Master Mix 15 µL, Bar-PCR primer F (10 μM) 1 μL, Primer R (10 μM) 1 μL, finally made up to 30 µL with water. The amplification program was as follows: 94 °C, 3 min; 94 °C for 20 s, 55 °C for 20 s, 72 °C for 30 s, 5 cycles; then 72 °C for 5 min.

The PCR products were purified using Agencourt AMPure XP beads (Beckman, Brea, CA, USA). A volume of magnetic beads that was 0.6 times the volume of the 25 μL PCR product was added and mixed well. The tube was placed on a magnetic stand for 5 min, and then the supernatant was carefully removed. A total of 30 μL wash buffer was added and mixed well. The tube was placed on a magnetic stand for 5 min, and then the supernatant was carefully removed. A total of 90 μL of 70% ethanol was added, and then the tube was placed on the magnetic stand to allow the magnetic beads to be adsorbed to the other side of the PCR tube. The supernatant was removed, and then the tube was placed in a 55 °C oven for 5 min. A total of 30 μL elution buffer was added, and then the tube was placed on a magnetic stand for 5 min. The supernatant was transferred to a clean 1.5 mL centrifuge tube. The recovered DNA was quantified using the Qubit 3.0 DNA assay kit (Life, USA). For sequencing, a total of 10 ng of DNA from each sample was taken, and the final DNA concentration for sequencing was 20 pmol.

### 4.3. Sequence Analysis and Taxonomic Identification of the Bacterial Community

Sample sequences were differentiated by barcodes. Firstly, the primers and adapter sequences were removed. Then, the paired reads are merged into a single sequence based on the overlap relationship between paired-end reads. Subsequently, quality control was applied to filter each sample sequence, removing nonspecific amplification sequences and chimeras to obtain valid data for further data analysis. Sequences were clustered based on distances, and then grouped into OTUs using cluster similarity at 0.97. Representative sequences were chosen from each OTU cluster based on their abundance, and alpha diversity analysis was performed using the OTU abundance data from each sample. Alpha diversity indexes, including Chao, ACE, Simpson, and Shannon were used, and coverage was calculated using Mothur (Version 1.48.1).

### 4.4. Pseudomonas Syringae Microbial Stress on SL

*S. stoloniferum* sample in this section was collected from the botanical garden of the Nanjing University of Chinese Medicine. The aboveground parts were trimmed, and healthy and uniform-sized rhizomes were selected and rinsed with tap water before being planted in nutrient soil in a cultivation water basin for germination. During this period, the water depth was maintained at 3–5 cm, and the plants were cultivated in a greenhouse with air temperatures ranging from 20 °C at 6 a.m. to 30 °C at 2 p.m. After germination, seedlings with similar growth were selected, transplanted into new pots, and cultivated for 35 days. The *P. syringae* pattern strain (strain number: SHBCC D10978; Shanghai Bioresource Collection Center, Shanghai, China) was cultivated on nutrient agar at 30 °C for 2 days until a bacterial lawn formed. The lawns were harvested and resuspended to an optical density of 0.2 at 600 nm. The bacterial solution was sprayed onto the soil of *S. stoloniferum* plants in the microbial stress group, and the SL sample was named PS. The negative control group was sprayed with water instead of *P. syringae* bacterial solution, and the sample was named BL. The rhizomes were collected 3 days after the initial infection.

### 4.5. LC–MS/MS Analysis of Microbial and Heat Stress SL

The PS and BL samples for LC–MS/MS analysis were the samples taken after microbial stress and non-stress treatment in Section 4.4; then, the *S. stoloniferum* rhizomes were cleaned with ddH_2_O, cut into 5 mm pieces, dried at 40 °C for 48 h, and ground into powder. The HT sample for LC–MS/MS analysis was *S. stoloniferum* collected from the botanical garden of the Nanjing University of Chinese Medicine. The aboveground parts were trimmed, and healthy and uniform-sized rhizomes were selected and rinsed with tap water before being planted in nutrient soil in a cultivation water basin for germination. During this period, the water depth was maintained at 3–5 cm, and the plants were cultivated in a greenhouse with air temperatures ranging from 20 °C at 6 a.m. to 30 °C at 2 p.m. After germination, seedlings with similar growth were selected, transplanted into new pots, and cultivated for 35 days in the greenhouse with air temperatures ranging from 20 °C at 6 a.m. to 30 °C at 2 p.m. *S. stoloniferum* plants in the heat stress group were cultivated in a greenhouse with air temperatures ranging from 25 °C at 6 a.m. to 40 °C at 2 p.m. *S. stoloniferum* plants in the negative control group were cultivated in a greenhouse with air temperatures ranging from 20 °C at 6 a.m. to 30 °C at 2 p.m. Then, the *S. stoloniferum* rhizomes were cut into 5 mm pieces, dried at 40 °C for 48 h, and ground into powder. An accurate weighing of 1 g solid sample was performed, followed by the extraction of metabolites using a solution (ethanol:water, 3:2, *v*/*v*). The mixture was ultrasounded at 40 kHz for 60 min. After centrifugation at 10,000× *g* at 4 °C for 15 min, the supernatant was filtered using a 0.22 μm nylon membrane. The supernatant and the supernatant diluted 20-fold were ready for LC–MS/MS analysis.

The LC–MS/MS analysis method was the same as in Section 4.1. Data were analyzed using SCIEX OS-Q software (Version 1.4.0.18067). Three biological replicates were used. ANOVA was conducted for comparison of groups and a *p* value ≤ 0.05 was regarded as statistically significant. Statistical analyses were performed, and graphs were generated using GraphPad Prism software (version 9.0).

### 4.6. RT–qPCR Analysis of Microbial Stress SL

The PS and BL samples for LC–MS/MS analysis were the samples taken after microbial stress and non-stress treatment in Section 4.4; then, the *S. stoloniferum* rhizomes were cleaned via ddH_2_O and cut into 5 mm pieces for RNA extraction. RNA extraction, cDNA synthesis, and RT–qPCR of SL samples under microbial stress group and negative control group were performed, and the primers and methods were the same as those described in the literature [22].

### 4.7. Identification and Enzymatic Activity Assay of Recombinant Protein Ss4CL

*Bam*H I and *Eco*R I restriction enzyme sites on both the *Ss4CL* gene-coding region and the pET28a expression vector were analyzed using SnapGene software (version 4.2.4). The selected restriction enzyme sites were used for vector construction, and the *Ss4CL*-CDS primer pair sequences are listed as follows: *Ss4CL*-CEF—CAGCAAATGGGTCGCGGATCCATGGGTTCGGTTAATTCGCC and *Ss4CL*-CER—TTGTCGACGGAGCTCGAATTCTTAAGCTGTAAGTCTTGCTCTCAGATC. The pET28a-*Ss4CL* recombinant vector was transformed into *Escherichia coli* DH5α competent cells (Tsingke, Beijing, China). Positive colonies were confirmed by PCR and sequenced by Shanghai Sangon Biotech Co., Ltd., Shanghai, China. Then, the pET28a-*Ss4CL* recombinant vector was transformed into *E. coli* BL21 (DE3) chemically competent cells (Tsingke, China) to induce expression. Protein expression was analyzed using 8% sodium dodecyl sulfate–polyacrylamide gel electrophoresis. The recombinant Ss4CL protein was purified using a BBI Ni-NTA gravity column (Sangon, Shanghai, China), BBI binding/wash buffer (Sangon, China), and BBI elution buffer (Sangon, China). The protein concentration was determined using a BCA protein quantitation assay kit (KeyGEN, Nanjing, China).

The standard substance of *p*-coumaryl-CoA (Lot number: A14IS222210) was purchased from Shanghai Yuanye Biotechnology Co., Ltd., Shanghai, China. Information on the *p*-coumaric acid standard substances is shown in Table S5. In 100 mM Tri-HCl buffer (pH 7.4), 100 mM MgCl_2_, 100 mM ATP, 10 mM CoA, different *p*-coumaric acid concentrations were added as substrates, and approximately 10 μg of recombinant Ss4CL were added to reach a total volume of 1 mL. The reaction was carried out at 45 °C for 30 min, and absorbance was measured at 334 nm using an ultraviolet–visible spectrophotometer to calculate *K*m, *V*max, *K*cat, and *K*cat/*K*m. The products from enzymatic reactions using the recombinant Ss4CL proteins and two negative controls, including the products from enzymatic reactions using the boiled recombinant Ss4CL proteins and the products from enzymatic reactions using the pET28a vector, were analyzed by an instrument platform for quantitative LC–MS/MS analysis, consisting of an ExionLC™ AD UHPLC System (AB Sciex, Framingham, MA, USA) and an AB Sciex Qtrap 5500 (AB Sciex, USA). Samples were separated using a ZORBAX Eclipse Plus C_18_ column (2.1 mm I.D. × 50 mm, 1.8 μm) and analyzed by mass spectrometry. The mobile phase consisted of methanol (solvent A) and 0.1% formic acid in 5 mM ammonium acetate (solvent B). To equilibrate the systems, the solvent gradient was changed according to the following conditions: from 0–0.5 min, 90% B; from 0.5–3.5 min, 90% B to 0% B; from 3.5–4 min, 0% B; from 4–4.1 min, 0% B to 90% B; from 4.1–5 min, 90% B. The sample injection volume was 5 µL and the flow rate was set to 0.3 mL·min^−1^. The column temperature was maintained at 40 °C. All samples were stored at 4 °C during the analysis period. Mass spectrometric data were collected in negative ion mode. Mass spectrometric data were collected in positive and negative ion modes. The optimal positive conditions were set as follows: CUR 10 psi, TEM 400 °C, GS1 30 psi, GS2 30 psi, ISVF 4500 V, CAD 10 psi, EP 10 V. The optimal negative conditions were set as follows: CUR 10 psi, TEM 400 °C, GS1 30 psi, GS2 30 psi, ISVF −4500 V, CAD 10 psi, EP −10 V. The optimized mass spectrum parameters of *p*-coumaric acid were shown in Table S8. For *p*-coumaryl-CoA, Q1 Mass was 914.7 Da, Q3 Mass was 407.3 Da and 428.1 Da, DP was 150 V, CE was 42.85 V and 42.92 V, and CXP was 15 V. Data were analyzed using SCIEX OS-Q software (Version 1.4.0.18067). Three biological replicates were used.

### 4.8. Indentication and Enzymatic Activity Assay of Recombinant Protein SsC4H and SsF3′5′H

*BamH* I and *EcoR* I restriction enzyme sites on both the *SsC4H* and *SsF3′5′H* gene-coding region and the pYedp60 expression vector were analyzed using SnapGene software. The selected restriction enzyme sites were used for vector construction and the *SsC4H*-CDS primer pair sequences and *SsF3′5′H*-CDS primer pair sequences are listed as follows: *SsC4H*-CEF—ACACACTAAATTACCGGATCCTTATCTATAGCCTTGGACTAGGTAGTAGTAGT, *SsC4H*-CER—TGGGAGATCCCCCGCGAATTCATGGATCTCCTCCTCCTCGAGA, *SsF3′5′H*-CEF—ACACACTAAATTACCGGATCCGCTGATAGACTTTATTTAATATAGGATTTATTCA and *SsF3′5′H*-CER—TGGGAGATCCCCCGCGAATTCTCATTCATAAGCTGCACGCACA. PCR amplification was performed using SL cDNA as a template. After detection by 1% agarose gel electrophoresis, the PCR product was recovered and ligated into the pCE2-TA Blunt-Zero vector (Vazyme, Nanjing, China). The recombinant plasmid was transformed into *E. coli* DH5α competent cells (Tsingke, China). Positive colonies were confirmed by PCR and sequenced by Shanghai Sangon Biotech Co., Ltd. After confirming the sequence, the pYedp60-*SsC4H* recombinant vector and pYedp60-*SsF3′5′H* recombinant vector was transformed into WAT11 chemically competent cells to induce expression, respectively.

Here, *trans*-Cinnamic acid standard substance (Lot number: Y11M10C88100) was purchased from Shanghai Yuanye Biotechnology Co., Ltd. Information on apigenin, luteolin, naringenin, eriodictyol, and *p*-coumaric acid standard substances are shown in Table S5. Then, *trans*-Cinnamic acid was added into pYedp60-*SsF3′5′H*-WAT11 bacterial solution as a substrate up to a final concentration of 0.5 mM. Naringenin and apigenin were added into pYedp60-*SsF3′5′H*-WAT11 bacterial solution as substrates to a final concentration of 0.5 mM, respectively. The reaction was carried out at 16 °C and 150 rpm for 12 h. The products from enzyme activity reactions using the recombinant target proteins and two negative controls including the products from enzymatic reactions using the boiled recombinant target proteins and the products from enzymatic reactions using pYedp60 vector were analyzed by an instrument platform for quantitative LC–MS/MS analysis consisting of an ExionLC™ AD UHPLC System (AB Sciex, USA) and an AB Sciex Qtrap 5500 (AB Sciex, USA). Samples were separated using a ZORBAX Eclipse Plus C_18_ column (2.1 mm I.D. × 50 mm, 1.8 μm) and analyzed by mass spectrometry. The mobile phase consisted of methanol (solvent A) and 0.1% formic acid in 5 mM ammonium acetate (solvent B). To equilibrate the systems, the solvent gradient was changed according to the following conditions: from 0 to 1 min, 95% B; from 1 to 4 min, 95% B to 5% B; from 4 to 4.1 min, 5% B to 95% B; from 4.1 to 6 min, 95% B. The sample injection volume was 5 µL and the flow rate was set to 0.3 mL·min^−1^. The column temperature was maintained at 40 °C. All samples were stored at 4 °C during the analysis period. Mass spectrometric data were collected in negative ion mode. Mass spectrometric data were collected in negative ion mode. The optimal negative conditions were set as follows: CUR 10 psi; TEM 400 °C; GS1 30 psi; GS2 30 psi; ISVF −4500 V; CAD 10 psi; EP −10 V. The optimized mass spectrum parameters of apigenin, luteolin, naringenin, eriodictyol, and *p*-coumaric acid were shown in the Table S8. Data were analyzed using SCIEX OS-Q software (Version 1.4.0.18067). Three biological replicates were used.

### 4.9. Structure Prediction of Ss4CL, SsC4H, and SsF3′5′H and Molecular Docking Studies

The structures of Ss4CL, SsC4H, and SsF3′5′H were predicted using AlphaFold2. Molecular docking studies were performed using the BIOVIA Discovery Studio 2019 software. Briefly, the predicted structure model of Ss4CL, SsC4H, and SsF3′5′H output by AlphaFold2 was docked with *p*-coumaric acid (compound CID: 637542), *trans*-cinnamic acid (compound CID: 444539), and apigenin (compound CID: 5280443), respectively. The Ss4CL, SsC4H, and SsF3′5′H molecules were added with non-polar molecules, and the receptor, ligand, and binding site were defined. LibDock molecular docking was performed under the following conditions: the number of hotspots was set to 100; docking tolerance was set to 0.25; docking preferences were set to high quality; and the conformation method was set to fast.

## Figures and Tables

**Table 1:** Alpha diversity index of soil bacterial communities at the root zone of SL in different production areas (*n* = 3).

Sample	Simpson	Shannon	ACE	Chao1	Coverage
SDY	0.0032 ± 0.0003 ^a^	7.5832 ± 0.0200 ^a^	13,791.94 ± 275.33 ^a^	13,069.06 ± 337.52 ^a^	0.9388 ± 0.0027 ^a^
SHF	0.0017 ± 0.0002 ^b^	7.5307 ± 0.0542 ^a^	10,266.53 ± 372.31 ^b^	9868.76 ± 422.31 ^b^	0.9556 ± 0.0054 ^b^
SYY	0.0014 ± 0.0001 ^b^	7.8798 ± 0.0582 ^b^	13,280.41 ± 344.07 ^a^	12,551.63 ± 258.86 ^a^	0.9381 ± 0.0014 ^a^

^a,b^ Different symbols in the same column indicate statistically significant differences in indicators between different production areas (*p* < 0.01).

## Data Availability

Data are contained within the article.

## Supplementary Materials

The supporting information can be downloaded at: https://zenodo.org/records/11444863.
